# Distinct spatiotemporal activity in principal neurons of the mouse olfactory bulb in anesthetized and awake states

**DOI:** 10.3389/fncir.2013.00046

**Published:** 2013-03-28

**Authors:** David G. Blauvelt, Tomokazu F. Sato, Martin Wienisch, Venkatesh N. Murthy

**Affiliations:** ^1^Department of Molecular and Cellular Biology, Harvard UniversityCambridge, MA, USA; ^2^Harvard Medical SchoolBoston, MA, USA; ^3^Center for Brain Science, Harvard UniversityCambridge, MA, USA

**Keywords:** anesthesia, sniff dynamics, inhibition, calcium imaging, awake mouse

## Abstract

The acquisition of olfactory information and its early processing in mammals are modulated by brain states through sniffing behavior and neural feedback. We imaged the spatiotemporal pattern of odor-evoked activity in a population of output neurons (mitral/tufted cells, MTCs) in the olfactory bulb (OB) of head-restrained mice expressing a genetically-encoded calcium indicator. The temporal dynamics of MTC population activity were relatively simple in anesthetized animals, but were highly variable in awake animals. However, the apparently irregular activity in awake animals could be predicted well using sniff timing measured externally, or inferred through fluctuations in the global responses of MTC population even without explicit knowledge of sniff times. The overall spatial pattern of activity was conserved across states, but odor responses had a diffuse spatial component in anesthetized mice that was less prominent during wakefulness. Multi-photon microscopy indicated that MTC lateral dendrites were the likely source of spatially disperse responses in the anesthetized animal. Our data demonstrate that the temporal and spatial dynamics of MTCs can be significantly modulated by behavioral state, and that the ensemble activity of MTCs can provide information about sniff timing to downstream circuits to help decode odor responses.

## Introduction

Volatile odorants are sensed in mammals by olfactory sensory neurons (OSNs), which converge on structures called glomeruli in the olfactory bulb (OB) (Mombaerts, 2006; Sakano, 2010; Murthy, 2011). Here, olfactory information is processed by an intricate circuit of neurons, including several classes of interneurons, and the processed output is carried by mitral/tufted cells (MTCs) to a variety of cortical structures (Shepherd et al., 2004; Mori and Sakano, 2011; Murthy, 2011; Wilson and Sullivan, 2011).

Neural recordings have offered ample evidence that odor representation and processing in early neural circuits can be altered by behavioral state (Kay and Laurent, 1999; Murakami et al., 2005; Kiselycznyk et al., 2006; Rinberg et al., 2006; Doucette et al., 2007; Doucette and Restrepo, 2008; Guerin et al., 2008; Tsuno et al., 2008). The dynamics of inputs to glomeruli are altered in waking conditions, largely due to changes in sniffing and adaptation (Kepecs et al., 2007; Verhagen et al., 2007; Carey and Wachowiak, 2011; Wachowiak, 2011). Postsynaptically, the spontaneous firing of MTCs is much higher in awake animals compared to anesthetized ones (Rinberg et al., 2006). Interestingly, the activity of MTCs is tightly modulated by sniffing, and odor-evoked changes in MTC spiking often are apparent only when examined in the context of sniffing, especially when animals are awake (Macrides and Chorover, 1972; Cury and Uchida, 2010; Shusterman et al., 2011). At least some of the changes observed in the activity of MTCs in awake animals comes from altered sniff parameters (Carey and Wachowiak, 2011), but additional effects due to top-down circuit modulation remain to be investigated. There is substantial feedback from olfactory cortical regions and midbrain neuromodulatory centers to the OB (Price and Powell, 1970; Davis and Macrides, 1981; Luskin and Price, 1983; Kay and Laurent, 1999; Matsutani and Yamamoto, 2008), which are likely to be modulated in a state-dependent manner.

To date, state-dependent changes in MTC activity have been studied at a single neuron level using extracellular recordings (Rinberg et al., 2006; Tsuno et al., 2008). With the exception of a recent study (Kato et al., 2012), the modulation of population dynamics of MTC activity as a function of behavioral state has not been studied, although inputs to the OB have been imaged in awake rodents (Verhagen et al., 2007; Vincis et al., 2012). Imaging can also offer information not easily obtained with electrical recordings—for example, the extent of propagation of activity along the lateral dendrites of MTCs, which can influence and be influenced by granule cell activity (Margrie et al., 2001; Chen et al., 2002; Lowe, 2002). Indeed, a recent study noted that the activity of mitral cells and granule cells were inversely related, but the spatial and temporal dynamics were not examined (Kato et al., 2012).

We sought to investigate how the population activity of output neurons is different in anesthetized and awake conditions using gene-targeted mice in which all principal neurons in the OB express GCaMP2, a genetically-encoded calcium indicator (Diez-Garcia et al., 2005; Fletcher et al., 2009). By imaging population activity in head-restrained animals and monitoring their sniffing simultaneously, we directly compared the spatiotemporal dynamics of postsynaptic activity in the awake and anesthetized states.

## Materials and methods

### Subjects

For most of our experiments, we used Kv3.1-GCaMP2 mice, in which GCaMP2 expression is driven by a promoter fragment from the Kv3.1 potassium channel (Diez-Garcia et al., 2005). Previous studies have found that the expression of GCaMP2 in these mice is confined largely to mitral and tufted cells (Fletcher et al., 2009). Data from nine adult (50–120 days old) mice were used for analysis (six for widefield, three for multiphoton). We also used four adult (p60) OMP-synaptopHluorin mice (Bozza et al., 2004) for some experiments.

### Headplate and sniff cannula implantation surgery

The initial surgery consisted of two stages performed in one session. The first was to implant the headplate and the second to implant a cannula used for sniff measurements. Adult male Kv3.1-GCaMP2 mice (50–120 days old) were anesthetized with a Ketamine/Xylazine (100 mg/kg:10 mg/kg, i.p., Webster) mixture and set on a stereotactic mouse holder. Body temperature was monitored with an external probe placed under the abdomen and maintained at 37°C with a homeothermic heating blanket (507220F, Harvard Apparatus). The scalp was retracted to expose the skull. After thorough cleaning and drying of the skull, a thin layer of cyanoacrylic adhesive (Vetbond, 3M) was applied to the skull and surrounding skin. Finally, a custom cut titanium headplate (Wienisch et al., 2012) was attached using acrylic cement (Jet Repair, Lang Dental). After the acrylic adhesive hardened (approximately 20 min), the sniff cannula implantation surgery was started. The area of the skull above the right nasal cavity and immediately anterior to the head-plate was thinned and removed. The epithelium was then punctured with a thin needle. After widening the hole and drying the cavity by removing blood and mucus, a short piece of medical tubing (#425415, BD Intramedic) was placed on top of the skull, aligned over the hole, and fixed with the acrylic cement.

### Head-fixation training

To minimize motion artifacts during imaging sessions, animals were trained to remain on a custom-built head-fixation setup. Adapting the methods of Dombeck et al. (2007), animals were placed on a styrofoam wheel (8″ diameter, 4″ wide, Smoothfoam, Plasteel Corp). The center of the wheel was pierced with a 2 mm diameter steel rod acting as an axle, and this axle was mounted to posts damped by soft springs. This method offered the animals' forward and backward freedom of motion while preventing uncontrolled lateral motion that a styrofoam ball may have (Wienisch et al., 2012). Training was started 48 h after headplate implantation surgery. Animals were acclimated to being placed on the wheel for short periods of time for multiple sessions daily. To further enhance animal place preference, animals were water restricted when not on the wheel and given 10% sucrose water *ad libitum* while on the wheel.

### Cranial window surgery

Mice were acclimated on the wheel for a minimum of 1 week before their craniotomy surgery and imaging sessions. The animal was anesthetized as described above, and a craniotomy was made over the left OB using a dental drill, ensuring that the dura was not damaged. After removing a portion of the skull, the surface of the brain was kept moist with artificial CSF (135 mM NaCl, 5.4 mM KCl, 5 mM HEPES, and 1.8 mM CaCl_2_, pH 7.4). The cranial window was then covered with 1.2% agarose (in aCSF) and closed with a 5 mm diameter glass coverslip. Mice were given doses of the analgesic Buprenorphine HCl (0.5 mg/kg) as needed, and imaging was conducted the same day, and up to 11 days post-surgery.

### Odor delivery

For all imaging experiments, odor was delivered through a custom-built olfactometer controlled through custom-written LabView code (National Instruments). Fresh air, odor, and again fresh air were delivered for 10 s each, with a 60 s inter-trial interval. In a few experiments, odor stimulus was on for only 5 s. Where applicable, these instances are noted in figures by an appropriate time scalebar. The following odors and concentrations (% volume pure odor per volume of mineral oil) were used: isopropyl tiglate (1:100), ethyl valerate (1:100), valeraldehyde (1:100), ethyl butyrate (1:100), 4-heptanone (1:100), isoamylamine (1:100), methyl tiglate (1:100), heptanal (1:100), thiazole (1:100), ethyl propionate (1:100), ethyl tiglate (1:1000), ethyl tiglate (1:200), ethyl tiglate (1:100), ethyl tiglate (1:20), ethyl tiglate (1:10), isoamyl acetate (1:1000), isoamyl acetate (1:200), isoamyl acetate (1:100), isoamyl acetate (1:20), and isoamyl acetate (1:10).

### Sniff monitoring

Sniffing behavior was monitored with real-time measurement of intranasal pressure (Verhagen et al., 2007). The medical tubing attached to the surface of the skull was covered with another piece of medical tubing (#427435, BD Intramedic) such that the connection was airtight. This tubing was then coupled to a pressure transducer (CPXL04GF, Honeywell Inc.) via an 18 gauge-to-luer stub adapter and Tygon tubing (1/8″ ID, 1/4″OD, Saint-Gobain). The signal was amplified (DAM50, World Precision Instruments, Inc.) before being acquired with an analog-to-digital converter (USB-6009, National Instruments). Data was recorded using custom software written in LabView (National Instruments).

### Wide-field imaging

Ketamine-Xylazine (100 mg/kg: 10 mg/kg, i.p.) was used to anesthetize animals for the “anesthetized” imaging trials. The OB was visualized using a custom-built microscope outfitted with a 10X objective (UPLFLN 10X, Olympus America). GCaMP2 was imaged using excitation light from a blue LED (Luxeon V Star, 470 nm, Philips Lumileds) and a fluorescence filter set (HQ480/40×, Q505LP, HQ510LP, Chroma Technology Corp.). A CCD camera (Sensicam HP, Cooke Corp.), binned 8 × 8, was used to image the OB at 33 Hz. With the magnification and binning, the image resolution was 6.2 μm per pixel, with an overall image dimension of 990 × 790 μm. Image acquisition, synchronized to odor delivery, was performed through custom-written LabView (National Instruments) software.

### Multi-photon imaging

A custom-built two-photon (2P) microscope was used for multi-photon imaging experiments. The OB was imaged with a water immersion objective (20×, 0.95 NA, Olympus America) at 910 nm using a Ti: Sapphire laser (Mai Tai, Newport Corporation). Image acquisition, synchronized to odor delivery, was done through custom-written LabView (National Instruments) software. For most experiments, images were obtained with a pixel resolution of 1 μm, with frame rates of 4 Hz. For experiments that involved comparisons with sniffing (Figure 5C, for example), higher frame rates of 8 Hz were used, with a consequent loss in spatial resolution (2 μm).

### Overall timeline of experiments

Day 0: Surgery for head implant and sniff cannula (adult mice 50–120 days old)
Day 2: Start of head restrained training on wheel
Day 9: Cranial window surgery for imaging
Day 9–20: Imaging. (We noticed no difference in the data whether imaging occurred on day 9 or later).

### Data analysis

All data analysis was performed using custom scripts written in Matlab (Mathworks). Sniffing signals were processed by mean filtering the raw analog signal and finding the inhalation peaks based on thresholding the slope and raw signal. Each trial was individually examined, and false positives and negatives were corrected. From the sniff peak data, frequency could be calculated as the inverse of the time between each peak. Raw image data were aligned to sniff data using transistor-transistor logic (TTL) signals of exposure times from the camera that was simultaneously collected with sniffing signals. The raw image stacks were used to create ΔF (odor signal minus air signal) glomerular response maps. Time courses were extracted from the image stacks by selecting responding glomeruli as regions of interest (ROI) and computing the mean fluorescence of the ROI over the course of the trial. Many of the anesthetized odor responses contained spatially extensive signals, making it difficult to identify individual glomerular hotspots. To better isolate hotspots in anesthetized animals (for visualization), we applied a Gaussian spatial filter with a standard deviation of 50 μm to the response image and subtracted this out. Such filtering was not done when we specifically analyzed diffuse responses.

### Prediction analysis

Each sniff-triggered response (STR) was found by taking the fluorescence time course data surrounding each inhalation peak. All of the STRs were then averaged to derive the sniff-triggered average (STA), the shape of the generic response. This shape was then scaled to match the amplitude of the first STR of the odor period. This scaled STA became the predicted response evoked by a sniff, and at every sniff peak, the STA was added to the prediction time course. For added complexity of modeling, we added the decay in response as an additional parameter. The actual amplitude of each STR was divided by the amplitude of the first STR. This normalized relative amplitude was then used as a lookup table to adjust the individual STR amplitudes as a function of sniff number. Beyond 40 sniffs, there was large variability due to small number of events for averaging and we used an extrapolated linear regression as the decay function.

To compare the prediction algorithms, we used the Extra Sum of Squares *F*-test (Motulsky and Christopoulos, 2004). We calculated the mean sum of squares of the residuals for each trial. The sum of all the mean values became the total sum of squares. Degrees of freedom were calculated as the number of trials minus the number of parameters used.

### Global response patterns

Glomerular hotspots were chosen for each mouse if they responded to any of the odors in either the anesthetized or awake states. These hotspots were then used to create response vectors for each odor, consisting of the ΔF values for each of the hotspots. Whole-field correlation studies were done to more objectively analyze global patterns. Images were filtered with a circular averaging filter with a 5 μm radius. The mean ΔF images in both the anesthetized and awake states were compared by linear correlation. In order to account for misalignment of the images, 1600 different cuts were taken, representing 35 μm (20 pixel) shifts in each direction, left, right, up, and down. The best correlation was taken to be the best alignment. Positive controls were calculated by finding the correlations between trials of the same odor within the anesthetized and awake conditions. A negative control was calculated by averaging the correlations between the mean anesthetized ΔF image of a given odor and a mean awake ΔF image of a random different odor. The mean correlation coefficients from multiple experiments were compared using a Komogorov–Smirnov test.

### Diffuse response

To examine spatially diffuse responses we drew concentric rings of 1.7 μm thickness around the glomerular hotspot and calculated the ΔF within each annulus. Points in the concentric rings that overlapped other hotspots were excluded. The average ΔF was then plotted as a function of distance from the glomerulus. For each trial, an exponential function was fitted, and the decay constant, τ, was calculated. The average τ values for anesthetized and awake were compared using the Kolmogorov–Smirnov test. Diffuse responses were also assessed by taking a Fourier transform of the ΔF images for each odorant and calculating the power as a function of spatial frequency. The relative power in low frequencies (0–2.5 mm^−1^) compared to high frequencies (25–35 mm^−1^) was used as an index of diffuse responses in anesthetized and awake conditions.

### Awake trial variability

Responses for individual awake trials were calculated in the same way as those for the analysis of global response patterns (see above). Scatterplots were created by pairing every awake trial with a different trial of the same odor. The ΔF values for each glomerulus in the two trials were plotted against each other. Normalized scatterplots adjusted ΔF values by either subtracting or dividing by the ΔF of the whole bulb. Correlations of the scatterplots were compared using Fisher's *z*-test. Individual fluorescence time courses were adjusted by subtracting out the whole-bulb time course. The trial-to-trial variability was first measured by calculating the average standard deviation between trials at each point in the time course. The average standard deviations were compared using the Kolmogorov–Smirnov test. Trial variability was also compared by plotting the ΔF values of individual trials vs. the mean ΔF value of the odor period across all trials.

Average data throughout the paper are reported as mean ± SD unless otherwise noted. The sample number refers to number of animals unless noted otherwise, often with data recorded from multiple sessions.

## Results

We used the Kv3.1-GCaMP2 mice because it was the most appropriate gene-targeted mouse line available at the time this study was initiated. Previous studies indicated that expression of GCaMP2 is largely confined to excitatory neurons—mitral and tufted cells (Fletcher et al., 2009). In the glomerular layer, ET cells are the main cell types expressing the indicator and none of the GABAergic neurons (identified with GAD67 staining) express GCaMP2 (Fletcher et al., 2009). Therefore, fluorescence signals recorded in wide-field microscopy arises mainly from principal cells in the olfactory bulb, with potentially some contamination from GAD65-expressing (Kiyokage et al., 2010) or unidentified non-GABAergic neuron in the glomerular layer.

### Dynamic sniffing behavior in awake mice

**Figure 1:**
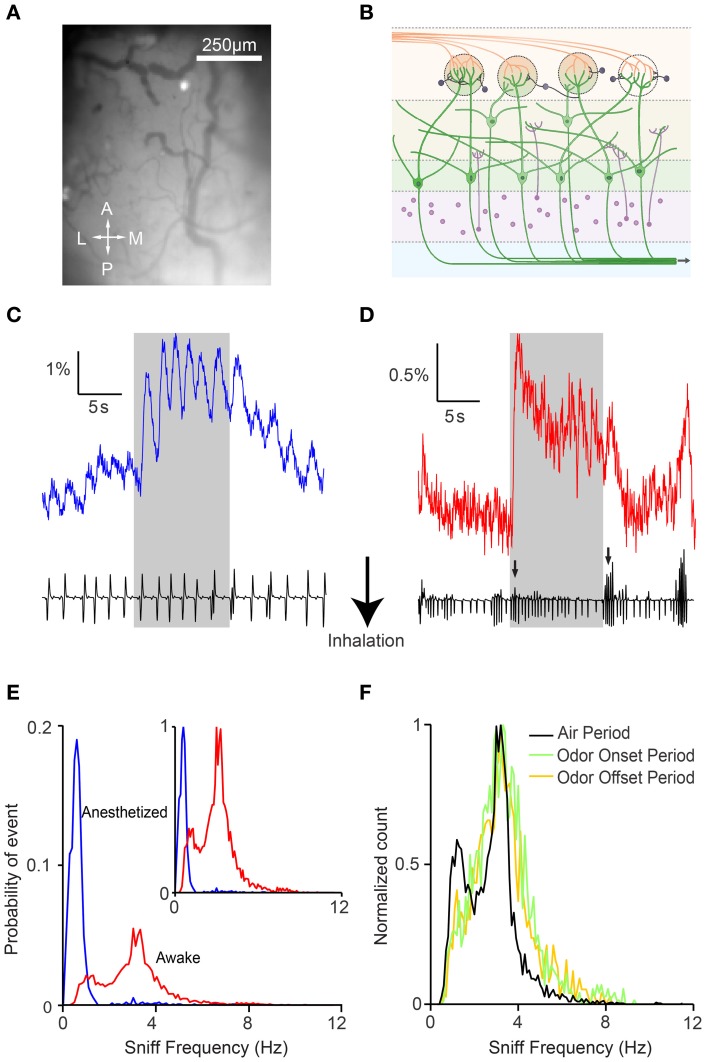
**Awake GCaMP2 mice display dynamic sniffing behavior compared to anesthetized mice. (A)** Average-intensity image of the olfactory bulb in the absence of odor stimulation. A, anterior; L, lateral; M, medial; P, posterior. **(B)** Schematic showing the expression of GCaMP2 in M/T cells. **(C)** Fluorescence time course of a single glomerulus in an anesthetized mouse with the corresponding nasal air pressure tracing. Gray shaded area represents the period of odor stimulation. **(D)** Fluorescence time course and sniff tracing for an awake mouse. Arrows indicate sniff bursts immediately after odor onset and offset. **(E)** Distribution of sniff frequencies in anesthetized (blue) and awake (red) mice. Inset depicts the same histogram normalized to the peak frequencies. **(F)** Normalized distribution of sniff frequencies in awake mice during the air period (black), odor onset period (green), and odor offset period (yellow).

We imaged post-synaptic neural responses and simultaneously recorded respiratory behavior in anesthetized and awake head-fixed GCaMP2 mice during passive odor delivery (Figure 1). Mice were trained to tolerate the head-fixed setup (Wienisch et al., 2012), but odors were not associated with a task or reward. Anesthetized mice displayed slow and regular respiration patterns accompanied by individually discernible fluorescence changes (Figure 1C). By contrast, awake mice had rapid, irregular respirations with less distinct individual responses (Figure 1D). In line with previous studies, we found that respiration in the anesthetized state was regular, with frequencies falling within a narrow range (0.79 ± 0.81 Hz, *n* = 6 animals) (Figure 1E).

Respiration in awake mice (sniffing) occurred at higher frequencies than in anesthetized mice, and the distribution covered a much larger range (Figure 1E; 2.81 ± 1.30 Hz, *n* = 6 animals). Interestingly, awake mice displayed a bimodal distribution of frequencies with peaks at 1.25 and 3.15 Hz. The faster sniffing frequency in our head-restrained preparation was lower than those seen in freely moving animals (Kepecs et al., 2007; Wesson et al., 2008b), but similar to that reported in other studies of head-restrained mice (Shusterman et al., 2011). We also noticed that mice tended to display more vigorous sniffing behavior during the first 2 s after the beginning and end of the odor presentation (Figure 1C, arrows), perhaps due to increased exploratory behavior upon introduction or cessation of novel stimuli (Kepecs et al., 2007; Wesson et al., 2008b). Indeed, while the distribution of sniffing behavior during the air period remained bimodal, respiratory patterns during the onset and offset were shifted toward higher frequencies (Figure 1F). In addition, although we were unable to compare absolute sniff amplitudes due to measurement variability, we were able to look for patterns in the relative changes in sniff amplitude. We found that the sniff amplitude was larger at odor onset (1.24 ± 0.51 relative increase versus air period, *n* = 6 animals, *p* < 0.001, two-sample *t*-test) and offset (1.27 ± 0.49 vs. clean air period, *p* < 0.001, two-sample *t*-test) in awake animals. Not surprisingly, respiration amplitude was not modulated systematically in anesthetized animals, and sniff amplitude was more variable in awake animals (standard deviation of 0.21 and 0.41 for anesthetized and awake mice respectively, *p* < 0.001, two-sample *F*-test).

### Sniff behavior is reflected in the fluorescence signal

**Figure 2:**
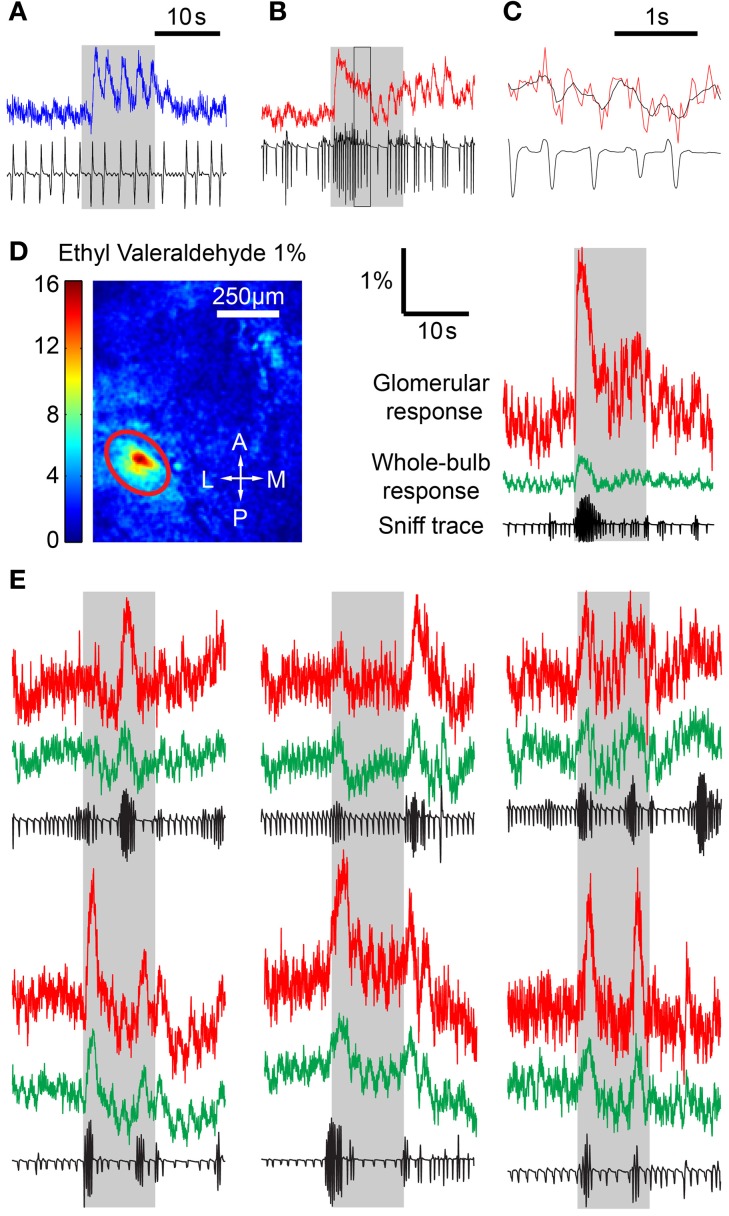
**Sniff behavior has an effect on odor responses. (A)** Time course of fluorescence in the OB in an anesthetized mouse with corresponding sniff trace, showing individual fluorescence fluctuations in response to a sniff. **(B)** Awake mouse time course. Individual sniff triggered responses are less easily identified. **(C)** Expanded version of the boxed region in **(B)** with the original fluorescence values (red) and a filtered time course (black). Fluorescence signal was filtered to remove high frequency noise. **(D)** Left, ΔF image from stimulation with 1% ethyl valeraldehyde. Responding glomerulus is circled in red. Right, time course plots from the encircled glomerulus (red) and the whole bulb (green) with corresponding sniff traces. Sniff bursts are correlated with response spikes across the whole bulb, including both responding and non-responding areas, even during clean air delivery. **(E)** Time courses and sniff traces from awake mice depicting the effect of sniff bursts on glomerular activity.

We next looked to see how differing respiratory behaviors affected the fluorescence responses from MTCs. In anesthetized animals, each inhalation induced an unambiguous response with complete or near complete recovery between breaths (Figure 2A). In awake mice, the sniff-locked response was less obvious, but despite a lower signal to noise ratio, individual modulations could be recognized even during periods of rapid sniffing (Figures 2B,C). Initial experiments indicated that odor stimulation led to focal increases in fluorescence, as well as more diffuse changes across large regions of the dorsal surface. We noticed that the response signals averaged over the entire imaged region of the OB showed clear modulation with respiration, and we analyzed these signals first (Figure 2D). Examination of the fluorescence traces revealed that sniff bursts in awake mice were clearly associated with increased fluorescence activity (Figures 2D,E). Intriguingly, rapid sniffing could induce clear fluorescence rises even during clean air delivery. To ensure that the whole-bulb responses were not simply contaminations of glomerular odor responses, we also excluded odor response hotspots and noted similar modulation with respiration in “non-responding” areas (data not shown). These modulations were not simply broad fluctuations related to hemodynamics (Verhagen et al., 2007), because they were clearly aligned to respiration. In addition, analysis of similar fluorescence changes in the OMP-spH animals revealed much slower sniff-burst-induced changes, as expected from a much slower reporter of presynaptic vesicle traffic. These data indicate that the modulation of fluorescence we observe is a function of the actual activity reporter (GCaMP2 or spH) rather than some other indirect index of activity such as hemodynamics or movement during sniffing.

**Figure 3:**
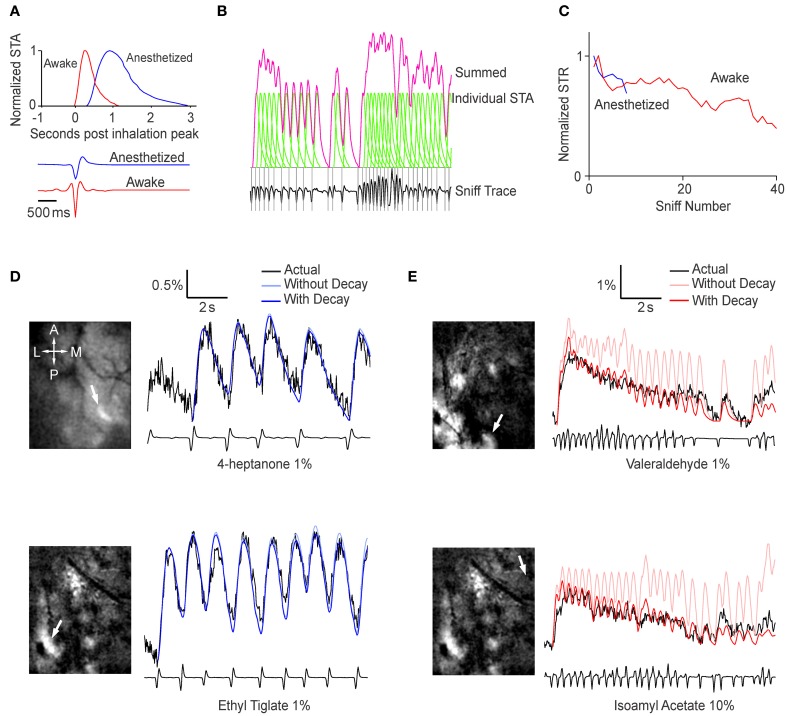
**Sniffing behavior can be used to predict the glomerular response. (A)** Top, normalized sniff-triggered average representing the generalized shape of the glomerular response to a single sniff in an anesthetized (blue) and awake (red) mouse. Bottom, average sniff traces showing the general shape of a single sniff in the two conditions. **(B)** Generation of the prediction time course. At each peak in inhalation (gray lines), the sniff-triggered average (green) is added to a baseline value. The sum of all the sniff-triggered averages is the prediction time course (pink). **(C)** Sniff triggered response amplitude as function of sniff number. Values are relative to the amplitude of the first sniff-triggered response and normalized to the peak sniff-triggered average. These values were used as a lookup table adjust the sniff-triggered averages **(D,E)**. Examples of prediction time courses for anesthetized **(D)** and awake **(E)** animals. The images on the left are ΔF projections with white arrows indicating the glomerulus used to derive the time courses.

A simple explanation for the increased post-synaptic activity during sniff bursts is temporal summation of fluorescence signals, which do not return to baseline in the short time between sniffs. We set up a simple prediction algorithm to test this idea. Using all trials (*n* = 6 animals, >4 sniffs per animal in anesth, >30 sniffs per animal in awake), we generated a normalized STA that represented the average fluorescence response evoked by a single sniff, as well as the average sniff shape (Figure 3A). We refer to breaths taken in the anesthetized condition as sniffs to simplify the language when referring to STAs. Interestingly, the shape of the breaths was different in awake and anesthetized animals—inhalation occurred first in anesthetized animals, but a brief exhalation preceded inhalation in awake animals. Because awake mice tended to sniff rapidly, the raw STA was contaminated by subsequent sniffs and did not fully recover to baseline. Therefore, we used a simple exponential extrapolation to fully account for return to baseline. We then used the STA to predict fluorescence changes based on the timing of sniff (Figure 3B). The simplest fitting algorithm we used involved a single amplitude variable that matched the amplitude of the first STR after odor onset, and remained the same throughout the odor period. The temporal summation of all the STRs yielded the predicted time course shown in Figure 3B. Examination of the fluorescence traces made it clear that a single amplitude value throughout the odor period could not describe the habituating fluorescence amplitudes. We empirically obtained the relative amplitude of the STR as a function of sniff (or breath) number (Figure 3C). We used this relation between amplitude and sniff/breath number as a lookup table to adjust the amplitude of the predicted fluorescence response to each sniff/breath.

Fluorescence changes in anesthetized animals were predicted well by a simple algorithm with a single value for the amplitude of STR (Figure 3D). Adjusting for habituation resulted in a statistically significant (*p* < 0.001, Extra sum of squares *F*-test, *n* = 4 animals) but small change (weighted sum of squares of the residuals = 0.195 without habituation parameter, 0.192 with habituation parameter). This is presumably because breaths were spaced well apart and there were too few breaths in an odor period to have any significant habituation. In awake animals, however, the prediction algorithm with single, fixed amplitude was less accurate (Figure 3E). The predicted responses tended to overestimate the actual time course, especially later in the odor period, but this was drastically improved by the empirically-derived decay parameter (Weighted sum of squares = 0.509 vs. 0.387 with habituation parameter, *p* < 0.001, *n* = 4 animals).

We chose to use sniff number as the independent variable for the sake of simplicity, but previous studies have suggested that neural activity in the OB may be attenuated during periods of high frequency sniffing (Verhagen et al., 2007). Adding sniff frequency to the analysis was technically challenging because of large variability across sessions, which led to a highly variable relation between inter-sniff interval and fluorescence response amplitude. Therefore, we acknowledge the effect of sniff frequency, but were unable to quantitatively account for it.

Taken together, these results indicate that even though the signals recorded in the awake mouse are highly dynamic, much of the fluctuation can be attributed simply to sniff behavior and habituation.

### Spatial pattern of activity in awake and anesthetized mice

**Figure 4:**
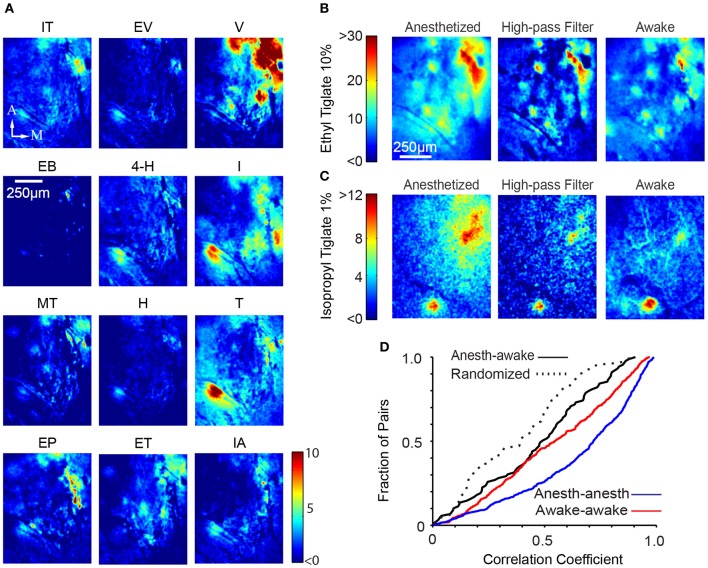
**Global glomerular patterns are conserved across behavioral states. (A)** Different odors show different response patterns. The images shown are ΔF images of the response of a single awake mouse to 12 different odors, averaged over three trials. IT, Isopropyl Tiglate; EV, Ethyl Valerate; V, Valeraldehyde; EB, Ethyl Butyrate; 4-H, 4-Heptanone; I, Isoamylamine; MT, Methyl Tiglate; H, Heptanal; T, Thiazole; EP, Ethyl Propionate; ET, Ethyl Tiglate; IA, Isoamyl Acetate; M, medial, A, anterior; Scalebar, 250 μm. **(B)** Glomerular activation pattern in an anesthetized (left) mouse and awake (right) after presentation of 10% ethyl tiglate. To more clearly distinguish individual hotspots in the anesthetized animal, we filtered the anesthetized image with a spatial high-pass filter (middle). Color bar indicates ΔF. **(C)** Second example of the similarity between anesthetized and awake activation patterns. These images were taken from a different mouse exposed to 1% isopropyl tiglate. **(D)** Distribution of correlation coefficients obtained from pair-wise comparison of spatial pattern of responses in different conditions.

We next sought to analyze spatial patterns of responses. As expected from many previous studies of the inputs to the OB, different odors evoked different glomerular response patterns (Figure 4A; awake responses). As noted above, odor responses had focal as well as diffuse components. By removing low spatial frequency components (see Materials and Methods), we were able to highlight glomerular-scale responses (Figure 4B).

We compared the spatial pattern of activity to the same odors in the anesthetized and awake animals. Visual inspection suggested that the activation patterns were largely similar (Figures 4B,C). We quantified the similarity of spatial pattern of responses in the anesthetized and awake conditions by performing cross correlation analysis (using mean subtracted Pearson correlation). For each odor, a single image of the response was generated by taking the difference between the odor and the preceding control period. The correlation coefficient between the corresponding response images in the anesthetized and the awake state was obtained (Figure 4D). To account for uncontrolled shifts in the imaging fields, we digitally shifted the images up to 35 μm and obtained the maximal correlation value. The average correlation coefficient between anesthetized and awake response patterns for the same odor was 0.47 ± 0.26 (*N* = 89 pairings, *n* = 6 animals). For comparison, we also obtained within-condition correlations by splitting trials of the same odor and condition into two groups. This value averaged 0.65 ± 0.28 (*N* = 645 pairings, *n* = 6 animals) and 0.55 ± 0.28 (*N* = 645 pairings, *n* = 6 animals) in anesthetized and awake animals respectively, which sets the maximum value that could be achieved when correlating anesthetized and awake trials, given the experimental variability. Both these values were significantly higher than the awake-anesthetized correlation (*p* < 0.001 and *p* < 0.05 respectively, Kolmogorov–Smirnov test). We also calculated the lower bound by correlating randomized trials across anesthetized and awake conditions (that is, not odor matched), which averaged 0.38 ± 0.24 (*N* = 89 pairings; Figure 4D) and significantly lower than the odor matched awake-anesthetized correlation value (*p* < 0.05). The non-zero correlation in the randomized trials is likely to be a result of our choice of related odorants. Furthermore, some trials tested the same chemical at different concentrations, further increasing the correlation value.

### Multi-photon imaging yields glomerulus-confined signals

**Figure 5:**
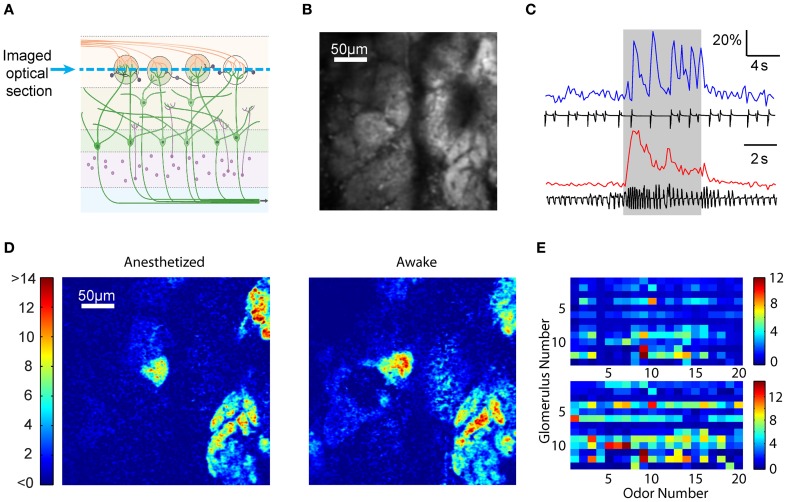
**Multi-photon optical sectioning confirms the consistency of glomerular activation across states. (A)** Schematic from Figure 1A depicting optical sectioning. Multi-photon microscopy excludes light from other layers, resulting in an image consisting only of a thin layer (blue dashed line). **(B)** Multi-photon image showing the resting activity of the glomerular layer. **(C)** Fluorescence time courses and sniff plots for an anesthetized (top) and an awake (bottom) mouse. Consistent with findings from wide-field microscopy, anesthetized animals show clear, sniff-locked responses, while awake animals display a noisier time course. **(D)** ΔF images from the OB of an anesthetized (left) and an awake (right) mouse exposed to 1% ethyl 3-hydroxybutyrate. Color bar indicates ΔF. **(E)** Response matrix of 13 glomeruli and 20 odors in an anesthetized (top) and an awake (bottom) mouse. Color bar indicates average ΔF in arbitrary fluorescence units. Note that while awake animals had larger glomerular responses, the activation patterns are similar.

Wide-field imaging, while technically easy, does not readily permit optical sectioning to obtain signals from a particular depth. To record glomerular signals in isolation, we used multi-photon microscopy in anesthetized and awake mice (Figure 5A) (Wienisch et al., 2012). Robust odor-evoked responses could be recorded in the glomerular layer from the M/T cell dendrites with a signal to noise ratio that was substantially better than that for wide-field imaging (Figures 5B,C). As observed with wide-field imaging, odor responses were clearly sniff-locked in the anesthetized preparation and more smoothed out in the awake brains (Figure 5C). We also noticed that the resting fluorescence in the glomerular layer was higher in awake than in anesthetized mice (1.14 ± 0.58 vs. 1.0 ± 0.65, *N* = 3 animals, *p* < 0.01, paired *t*-test), in line with the higher spontaneous activity in MTCs in the awake state (Rinberg et al., 2006). Individual glomerular responses to odors could be readily identified due to lack of spatial blurring (Figure 5D). Taking advantage of these demarcated responses, we compared the spatial pattern of activity in the awake and anesthetized states in the same animals. As shown in Figure 5E, the glomerular pattern of responses was quite similar in the two states. The correlation of the response pattern was 0.80 ± 0.15, which is slightly, but significantly (*p* < 0.01, Kolmogorov–Smirnov test) lower than the within state-correlation (anesthetized) of 0.95 ± 0.06 (*N* = 2 animals, 30 glomeruli).

Our analysis indicated that the spatial patterns of activity across behavioral states are significantly more similar than those across odors, but there are some differences. One such difference is discussed next.

### Spatially diffuse odor responses in anesthetized animals

**Figure 6:**
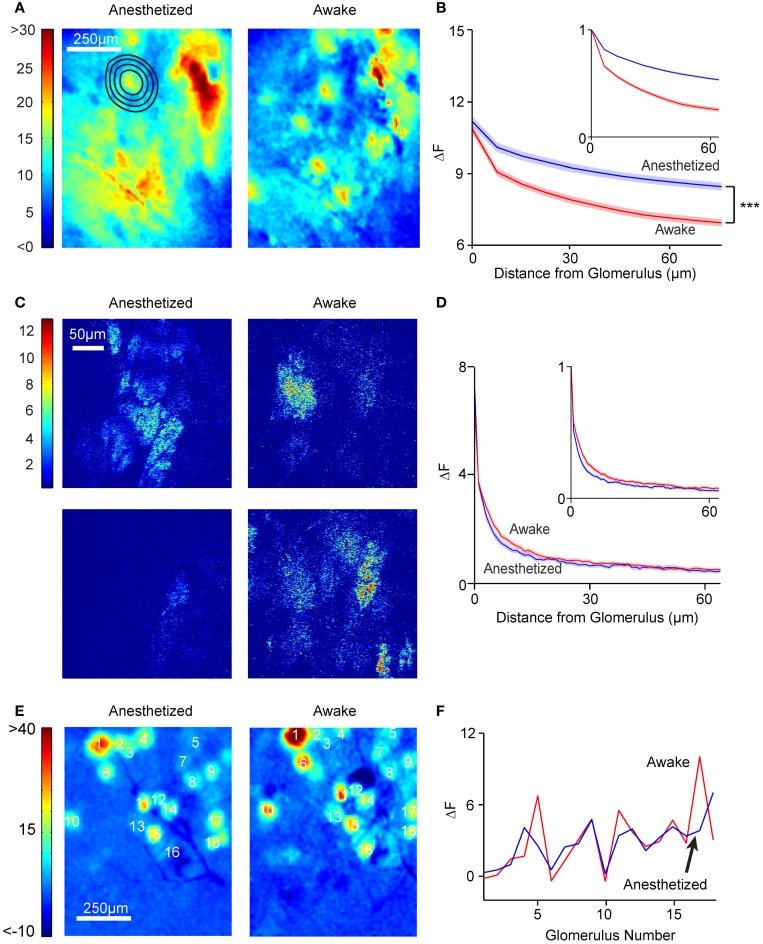
**Anesthetized, but not awake animals, have diffuse activation patterns. (A)** Widefield glomerular activation pattern in an anesthetized (left) and awake (right) mouse after presentation of 10% ethyl tiglate. Note that it is easier to distinguish individual activated glomeruli in the awake mouse. **(B)** ΔF as a function of distance from the glomerulus in anesthetized (*n* = 6 mice, 256 glomeruli) and awake (*n* = 6 mice, 196 glomeruli) mice. Inset, ΔF values are normalized to the ΔF of the glomerulus. Shaded areas denote standard error. **(C)** Response images (ΔF) obtained with multi-photon imaging in the glomerular layer. Note that neither anesthetized nor awake animals had significant diffuse responses. **(D)** ΔF values as a function of distance from the glomerulus. Blue, anesthetized (*n* = 6 mice, 77 glomeruli). Red, awake (*n* = 6 mice, 99 glomeruli). **(E)** Odor responses in OB of OMP-synaptopHluorin mice did not exhibit diffuse patterns. Shown are example global activation patterns in the same mouse in anesthetized (left) and awake (right) states. **(F)** Plot shows the ΔF values for the 18 labeled glomeruli in anesthetized and awake conditions.

Although the spatial pattern of responses to odors was generally similar in anesthetized and awake animals, we noticed the presence of a spatially “diffuse” response in anesthetized mice that was not as prominent in awake mice (Figure 6A). We quantified this diffuse response by selecting responding hotspots and calculating the average fluorescence changes in the regions surrounding the hotspots. This analysis indicated that fluorescence changes in areas outside of the responding glomerulus were larger in anesthetized animals, even when accounting for the increased glomerular response in anesthetized animals (Figure 6B; decay space constant of 326 ± 51.2 μm in anesthetized vs. 166 ± 32 μm in awake, *n* = 6 animals, 263 spots, *p* < 0.001, Kolmogorov–Smirnov test). The larger spatial spread of activation in the anesthetized animals was also observed when only isolated hotspots were considered (140.2 ± 12.8 μm vs. 76.2 ± 12.8 μm, *n* = 6 animals, 242 spots, *p* < 0.001 Kolmogorov–Smirnov test).

The above analysis relied on identification of focal responses and estimating the decay of surrounding fluorescence changes. To avoid any potential biases in identifying (or missing) focal spots, we also used a different method to estimate the extent of diffuse responses in the awake and anesthetized conditions. We reasoned that more diffuse responses should lead to a greater relative weight to low spatial frequencies in the response images. Therefore, we calculated the Fourier transform of the normalized response images and averaged the power (square of the amplitude) at each spatial frequency across multiple experiments in the anesthetized and awake conditions. On average, there was more power at low spatial frequencies in anesthetized animals compared to awake ones, with the ratio of power in high and low spatial frequencies averaging 0.25 ± 0.23 in anesthetized and 0.74 ± 0.39 in awake animals (*p* < 0.001; *N* = 60 odor responses from 3 animals). These data confirm the findings from the decay analysis above.

What could the source of the diffuse response be? A large part of the signal recorded in the wide-field imaging mode is likely to arise from the superficial layers, but signals from deeper layers [for example from the external plexiform layer (EPL)] will still reach the objective lens. To test whether the greater diffuse responses in the anesthetized state arise from the glomerular layer, we examined the data from multi-photon microscopy, which allowed us to obtain thin optical sections. As noted earlier, odor-evoked responses in the glomerular layer were spatially confined, and quantification revealed no difference (*p* > 0.05, Kolmogorov–Smirnov test) in the spatial decay constant between anesthetized (5.7 ± 0.5 μm) and awake (7.1 ± 0.5 μm) animals (*n* = 3) in the extent of spatial spread (Figures 6C,D). This argues against the possibility that the diffuse responses are simply a manifestation of a larger activation of more glomeruli in the anesthetized state.

The lack of a diffuse response in the glomerular layer is further supported by studies in mice expressing the presynaptic probe, synaptopHluorin, in OSNs (Bozza et al., 2004), in which responses had similar spatial patterns in awake and anesthetized states (Figures 6E,F). By elimination, our studies point to deeper layers as the source of diffuse responses—most likely, the lateral dendrites of M/T cells in the EPL. We were unable to obtain direct recordings of multi-photon responses in the EPL in awake mice due to large motion artifacts that affected imaging of fine M/T lateral dendrites much more than glomerular layer processes.

### Correcting trial-to-trial variability

**Figure 7:**
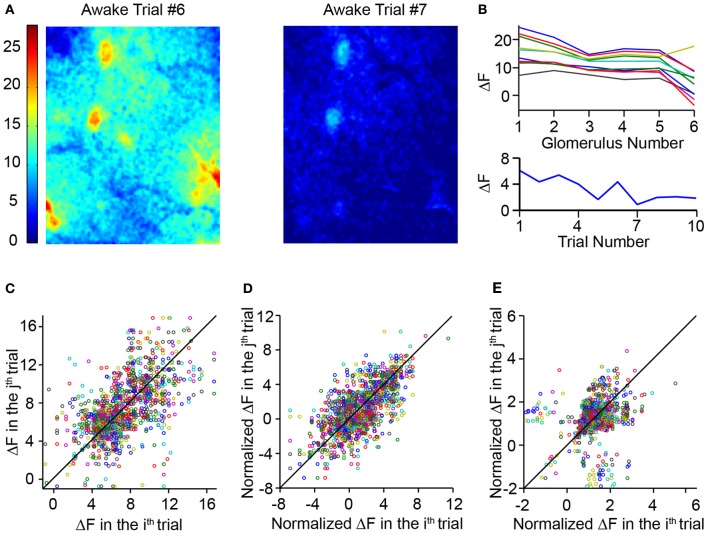
**Awake mice had highly variable responses. (A)** Glomerular activation pattern in an awake mouse evoked by 10% ethyl tiglate. Images represent two trials in the same mouse with the same odor. **(B)** Top, ΔF responses of six responding glomeruli over 10 different trials. Trials of the same odor were spaced approximately 9 min apart. Note that while the absolute ΔF values varied significantly, the relative values of the six glomeruli remained similar. Bottom, average whole-bulb response over the same 10 trials. While the average response decreases over the 10 trials, the fluctuation cannot be explained solely by habituation. **(C)** Scatterplot comparing the ΔF of a glomerulus in a single trial to the ΔF values of the same glomerulus in each other trial. **(D)** Scatterplot similar to c but comparing normalized ΔF values. ΔF was normalized by subtracting the average ΔF value of the entire dorsal bulb. **(E)** Scatterplot comparing ΔF values normalized by dividing ΔF values by the whole dorsal bulb ΔF.

The magnitude of responses to a given odor across multiple trials in the awake animal appeared to be highly variable (Figure 7A). This was also demonstrated earlier by the correlation analysis, in which response pattern correlations between awake trials were significantly lower than within anesthetized trials. However, when responses of different glomeruli across different trials of a single odor were plotted, it became clear that the variations in response amplitude were correlated across the glomeruli (Figure 7B). The overall global response fluctuates, but diminishes as a function of trial number (Figure 7B). This variation is not simply due to habituation because a given odor was presented with an inter-trial interval of around 9 min. The correlated trial-to-trial fluctuations in response amplitudes could arise either as a result of an underlying additive background signal or a multiplicative amplitude change. We tested each of these possibilities by obtaining the average ΔF value over the whole bulb and either subtracting or dividing this value from each glomerular response. First, a scatter plot of the response amplitude on a particular trial against response in another trial was created for all glomeruli and odors (Figure 7C). When a similar plot was obtained with responses adjusted by subtractive normalization, the scatter was significantly reduced (Figure 7D; correlation coefficient 0.67 for normalized plot vs. 0.58 for raw plot, *N* = 1400 trial pairings from 242 hotspots over 6 animals, *p* < 0.001, Fisher's *z*-test). Simple divisive normalization did not reduce variability (Figure 7E), and actually reduced the correlation (0.26; *p* < 0.001, Fisher's *z*-test when compared to raw plot).

Given our previous findings that the sniffing behavior can have significant effects on post-synaptic activity (Figure 3), it seems likely that the trial-to-trial variability was a direct effect of changing dynamics of sniffing. Downstream neural circuits, such as the olfactory cortex, must take into account sniffing to interpret changes in the activity of bulbar neurons—for example, to distinguish between a response due to an odor and a response generated simply by the act of sniffing. Such a “deconvolution” could be accomplished if these downstream areas receive exogenous information about sniff behavior (some corollary discharge from respiratory centers, for example). Alternately, sniff dynamics could be extracted directly from the responses of bulbar neurons. Because responses due to the act of sniffing would presumably affect both responding glomeruli and non-responding glomeruli equally, we supposed that cortical neurons could use average activity of the entire bulb as a proxy for sniffing behavior.

**Figure 8:**
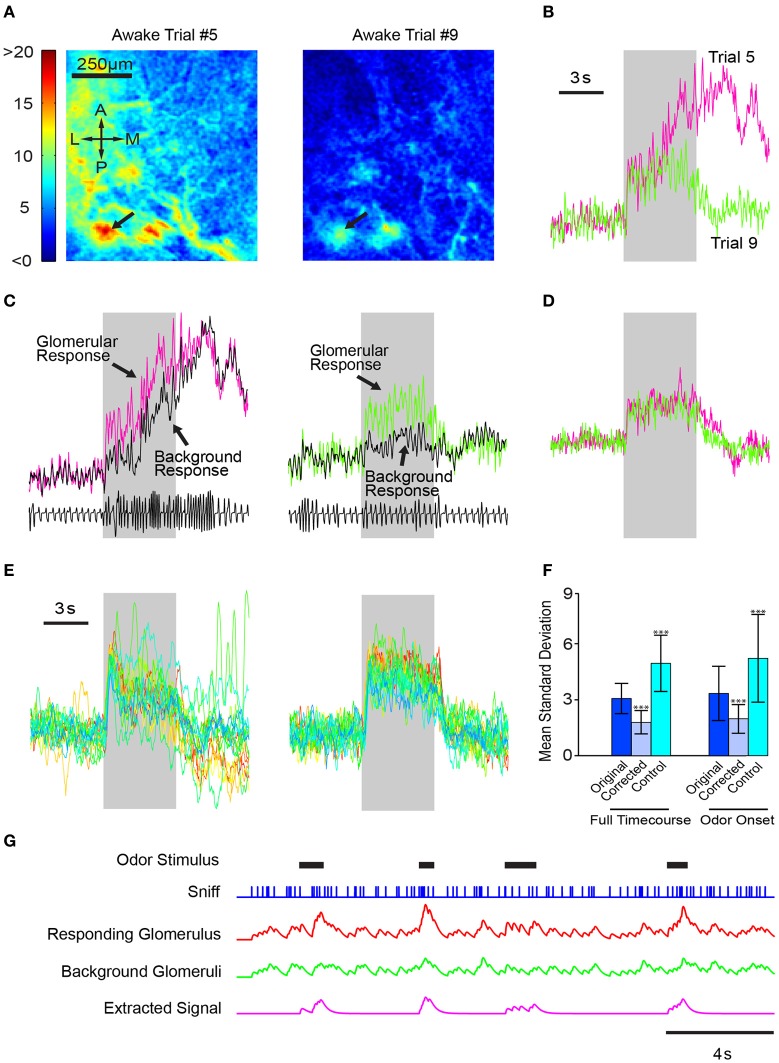
**Consistent response profiles in awake mice can be derived by subtracting whole-bulb responses. (A)** Glomerular activation profiles in an awake mouse during two different trials (5 and 9, respectively) of 1% valeradehyde presentation. **(B)** Response profiles of the glomerulus indicated by the arrows in **(A)**. **(C)** Glomerular response profiles plotted with the whole dorsal-bulb background response. **(D)** Corrected glomerular response time courses obtained by subtracting the background response from the glomerular response. **(E)** Left, glomerular responses of 20 different trials of the same odor. Right, time courses of the same 20 trials after background subtraction. **(F)** Bar graph depicting the average standard deviation of each point in the time course across all trials of the same odor (*n* = 6 mice, 79 total odors). Instead of subtracting the background response from the glomerular response, the control algorithm added the two time courses together. Errorbars indicate standard deviation. ^***^p < 0.001. **(G)** Illustration of the utility of removing global responses to better identify specific odor responses. Odors come on at random times, and sniffing frequency varies continuously. Each sniff produces a small response in all glomeruli when there is no odor; when a particular odor is present, a stronger response occurs in a glomerulus that is innervated by OSNs responding to that odor. Some odor responses can be detected by simple threshold crossing (the second and fourth odor response), but others become clearer when responses from background glomeruli are subtracted.

To test this model, we used a simple algorithm to adjust for sniff dynamics. We first derived a background time course using the fluorescence activity of the entire dorsal OB. We then subtracted the whole-bulb time course from the glomerular response and compared the corrected glomerular responses of different trials of the same odor. Even in examples with large variability amongst trials (Figures 8A,B), using the whole-bulb fluorescence time course as a proxy for sniff behavior significantly improved the consistency of an odor response (Figures 8C–E). We quantified the variability for the raw and corrected signals by taking the average standard deviation across different trials over the entire odor period. The standard deviation was 3.06 ± 0.80 for the raw traces and 1.81 ± 0.62 for the subtraction normalized traces (Figure 8F; *p* < 0.001, Kolmogorov–Smirnov test, *N* = 2 animals, 79 hotspots). This improvement was not simply the result of mathematical manipulation because adding the background signal instead of subtracting increased the variability (Figure 8F; standard deviation of 4.97 ± 0.1.42, *p* < 0.001 when comparing either raw or subtraction-normalized data). The reduction in trial-to-trial variability was observed irrespective of the time period considered—the entire odor period or just the odor onset (Figure 8F).

Our results demonstrate that using the whole-bulb “background” signal as a proxy for sniff behavior eliminates the need for a separate respiratory input to the olfactory cortical processing centers and allows the recovery of more consistent trial-to-trial olfactory signals. This is illustrated in a schematic model, where odors come on at unpredictable time and increase the activity by two-fold compared to sniffing clean air (Figure 8G). As illustrated, responses are not easily discerned in the noisy activity of the glomerulus that receives input from the cognate odorant receptor, but if sniff-locked activity from non-responding glomeruli is subtracted, responses become clear (Figure 8G).

## Discussion

We used population imaging to find that the spatial patterns of odor-evoked activity on the dorsal surface were generally similar in anesthetized and awake animals, but with some differences due to infra-glomerular signals that can likely be attributed to MTC lateral dendrites. The temporal dynamics of activation, on the other hand, were significantly different in the two conditions, in part due to variation in sniffing patterns. The highly variable sniffing pattern in awake animals led to significant disparity in the dynamics of fluorescence signals from MTCs over multiple trials. Relatively invariant odor-evoked responses could nevertheless be extracted from the data by removing the effects of sniffing, which could be obtained from the population signals in principal cells without any direct knowledge of sniff times.

### Sniff-locked responses in awake mice

Our recordings indicate that postsynaptic signals are clearly modulated by sniffing especially in awake mice. An earlier study reported that OSN input signals were smeared at high frequencies, and suggested that sniff-locked responses might be lost during fast sniffing (Verhagen et al., 2007). However, spike recordings from individual neurons (putative MTCs) have confirmed sniff-locking even at high frequencies (Cury and Uchida, 2010; Carey and Wachowiak, 2011; Shusterman et al., 2011). Our population-level imaging revealed widespread synchronization of neural activity by sniffing in the OB of awake mice, even at higher frequencies.

The average STR had a briefer time course in awake animals compared to anesthetized ones, likely due to shortening of response times during higher frequency of sniffing in awake animals. Indeed, recordings of single neurons in the anesthetized OB responding to “playback” of different sniff frequencies revealed tightening of response times at high frequencies (Carey and Wachowiak, 2011). One reason for this could be more rapid recruitment of inhibition during higher frequency sniffing (Balu et al., 2004; David et al., 2008; Carey and Wachowiak, 2011).

The temporal dynamics of evoked neural activity were highly variable in the awake animal, in contrast to the repeatable responses in the anesthetized state. Much of this variability was related to the fluctuations in sniffing rate. As noted before by others (Welker, 1964; Kepecs et al., 2007; Wesson et al., 2008b), sniffing occurs at higher frequency in awake animals and the frequency can be modulated rapidly. Sniffing did not follow stereotyped patterns seen in other studies, perhaps because odors were passively delivered without any specific task. Changes in overall neural activity could be observed even in the absence of odor stimuli, simply from bursts of high frequency sniffs. Similarly, even offset of odors could lead to apparent responses if sniff bursts occurred, and different trials of the same odor stimulus yielded highly differing responses. Because of this variability, odor-related neural activity can only be interpreted with knowledge of sniffing.

### Spatial pattern of activity in awake and anesthetized animals

A recent study using intrinsic optical imaging suggested that odors lead to denser activation in the glomerular layer in awake mice compared to anesthetized ones (Vincis et al., 2012). However, respiration was not measured in those experiments and it is possible that the stronger responses were due to increased rate of sniffing, which might lead to increased concentrations of odors in the epithelium as well as to greater temporal summation. Another study in anesthetized animals indicated that the spatial pattern of activity may vary with nasal airflow rate (Oka et al., 2009).

We directly measured neural signals while monitoring sniffing at the same time, and found that the spatial pattern of activity in the glomerular layer was largely similar in different states, despite the differences in the rates and amplitude of sniffing. Because sniffing is much faster in awake animals, larger transient changes occasionally did occur, though not affecting spatial pattern. Our findings suggest that there may not be significant differences in any lateral interactions that exist in the glomerular layer (Aungst et al., 2003; McGann et al., 2005; Vucinic et al., 2006) in the awake vs. anesthetized animals. Our data, however, do indicate that there may be significant differences in lateral interactions below the glomerular layer, as discussed below.

A recent study used cellular resolution imaging *in vivo* and found that fewer mitral cells are activated by odors in awake mice compared to anesthetized ones (Kato et al., 2012), but no information was provided about the extent of propagation along lateral dendrites. Imaging in the glomerular layer, that study also found that the spatial pattern of responses were similar in anesthetized and awake states, matching our findings.

### Greater lateral spread of activity in anesthetized animals

Although the spatial pattern of focal responses to odors were similar in the anesthetized and awake states, spatially diffuse responses were more prevalent in anesthetized state. This was evident with wide-field imaging, where signals are recorded from not just the glomerular layer. Optical sectioning using multi-photon microscopy revealed that odor-evoked responses respected glomerular boundaries in the glomerular layer. Therefore, the origin of the diffuse signals recorded in wide-field imaging must have been below the glomerular layer. It is unlikely that the diffuse fluorescence arises from the soma of MTCs for two reasons. First, fluorescence signals from very deep layers (mitral cell layer, for example) are unlikely to reach the surface given the scattering and absorption in the intervening layers. Second, because MTCs associated with an active glomerulus are likely to be located within a region below the glomerulus with a radius of around 150 microns (Buonviso et al., 1991), the diffuse responses cannot arise from spatially disperse somatic responses. Mitral cell lateral dendrites, however, are very long and can reach distances of 800 microns or more from the soma in rodents (Orona et al., 1984; Urban and Sakmann, 2002). Because activity can propagate along these secondary dendrites for long distances (Margrie et al., 2001; Chen et al., 2002), activation of even a single glomerulus can initiate laterally spreading activity. Thus, the most likely source of the diffuse signals is the EPL, made up largely of secondary dendrites of MTCs. In a different study from our group, we have imaged mitral cell and EPL activity using a large suite of odors and found that dendrites in the EPL were active even in the absence of mitral cell somatic responses immediately below (Albeanu and Murthy, unpublished data), supporting the idea of lateral propagation in MTCs.

What mechanisms could account for reduced diffuse activity in awake animals? Sniffing occurs more rapidly in awake animals, and it is possible that the reduced lateral spread is related to the higher sniffing rate. Faster sniffing may recruit greater glomerular inhibition by more potent activation of external tufted cells and subsequent recruitment of glomerular interneurons (Wachowiak and Shipley, 2006; Carey and Wachowiak, 2011). While this mechanism could explain the shorter time course of the STAs in awake animals, it is unlikely to fully account for the reduced lateral spread as it involves only intra-glomerular interactions that may reduce the overall activity of M/T cells, and does not easily explain the attenuation in lateral dendrites. Our data also argues against such overall reduction in activity playing a role in determining lateral spread because normalizing for response amplitudes did not alter the basic finding. In addition, multi-photon microscopy revealed little evidence for reduced overall postsynaptic activity in the glomerular layer in awake animals, and presynaptic activity imaged in OMP-spH mice was also not weaker in awake animals.

We favor an explanation for the reduced lateral spread of activity in awake animals that involves attenuated propagation along lateral dendrites, presumably due to increased inhibition from granule cells, which can shunt action potentials in lateral dendrites (Chen et al., 2002; Lowe, 2002). Odor-evoked granule cell activity may be different in awake and anesthetized animals (Kato et al., 2012), especially if cortical input to granule cells (Price and Powell, 1970; Davis and Macrides, 1981; Matsutani and Yamamoto, 2008; Restrepo et al., 2009; Boyd et al., 2012; Markopoulos et al., 2012) is state-dependent. A recent study indicated that granule cells are indeed more readily activated in awake animals (Kato et al., 2012), perhaps helping to shunt the propagation of activity in M/T secondary dendrites.

### Decoding odor responses without direct sniff information

In awake animals, different trials of the same odor stimulus gave rise to widely differing response profiles, largely due to variability in sniffing. Areas downstream of the OB will need information about sniff timing to decode MTC activity, and our studies indicate that the overall population activity can itself be used to infer sniff dynamics.

The population activity in the OB shows clear modulation with breathing/sniffing even in the absence of odor stimuli, in line with expectation from single neuron recordings (Macrides and Chorover, 1972; Onoda and Mori, 1980). In many sensorimotor systems, efference copies of motor commands are thought to be relayed to sensory areas as reference signals (Crapse and Sommer, 2008; Hill et al., 2011; Wurtz et al., 2011). In the OB, however, the respiration-coupled activity is likely to be driven by nasal airflow (Grosmaitre et al., 2007; Phillips et al., 2012) and not due to an efference copy of respiration signals from central areas (Phillips et al., 2012).

We found that any variability associated with fluctuating rate of sniffing can be removed by subtracting out population activity. If responses to odors are carried by a relatively sparse set of neurons, and a large population of neurons is modulated by sniffing, then an average of a random set of neurons should provide a reasonable proxy for breathing. Indeed, we found that a simple global average of bulbar activity can be subtracted from the individual glomerular response to reduce trial-to-trial variability. Physiologically, such a subtraction could be achieved by broadly tuned inhibition converging on principal cells, which has been recently described in the piriform cortex (Poo and Isaacson, 2009). Interneurons in many sensory cortical areas have broad tuning and are thought to provide forms of globally averaged inhibition to projection neurons. The removal of broadly correlated activity can act as a form of decorrelation that occurs in many neural circuits—here it might serve to remove fluctuations in neural activity arising simply due to sniff variability.

Such a referencing scheme could offer many biological advantages. Reference signals originating from the olfactory periphery, in contrast to those from central efferents, may be modulated by changes in temperature, humidity, or other nasal conditions in the same way as odor signals, allowing more accurate subtraction of these variables. For example, a scheme that uses bulbar neural activity itself could balance out signal strength from each naris and offer a coherent olfactory scene even in the face of changing differences in the condition of the two nares. Rodents have a remarkable ability to identify and discriminate between similar odors with a single sniff (Uchida and Mainen, 2003; Abraham et al., 2004; Wesson et al., 2008a). It will be interesting to determine whether such population activity is used to account for trial-to-trial variability in sampling, perhaps by combining optogenetic modulation of global activity levels in one or both bulbs and an odor discrimination task.

In summary, our study has revealed important differences in the spatial and temporal patterns of activity in OB principal cells in different behavioral states.

### Conflict of interest statement

The authors declare that the research was conducted in the absence of any commercial or financial relationships that could be construed as a potential conflict of interest.
