# Erratum: Distinct spatiotemporal activity in principal neurons of the mouse olfactory bulb in anesthetized and awake states

**DOI:** 10.3389/fncir.2013.00114

**Published:** 2013-06-26

**Authors:** David G. Blauvelt, Tomokazu F. Sato, Martin Wienisch, Thomas Knöpfel, Venkatesh N. Murthy

**Affiliations:** ^1^Department of Molecular and Cellular Biology, Harvard UniversityCambridge, MA, USA; ^2^Harvard Medical SchoolBoston, MA, USA; ^3^Center for Brain Science, Harvard UniversityCambridge, MA, USA; ^4^Division of Brain Sciences, Imperial College LondonLondon, UK; ^5^Laboratory for Neuronal Circuit Dynamics, RIKEN Brain Science InstituteWako-shi, Japan

Thomas Knöpfel, a key contributor to the work published recently in Frontiers in Neural Circuits (*Front. Neural Circuits* 7:46. doi: 10.3389/fncir.2013.00046) was inadvertently omitted in the list of authors.

We apologize to the readers for the inconvenience caused by this omission.

